# Supplementary material for: A De Novo Sequence Variant in Barrier-to-Autointegration Factor Is Associated with Dominant Motor Neuronopathy
Source: Cells. 2023 Mar 9;12(6):847. doi: 10.3390/cells12060847 (PMC10099716; doi:10.3390/cells12060847)
Supplement: Supplementary file 1 [file cells-12-00847-s001.zip › cells-2229868-supplementary.pdf]

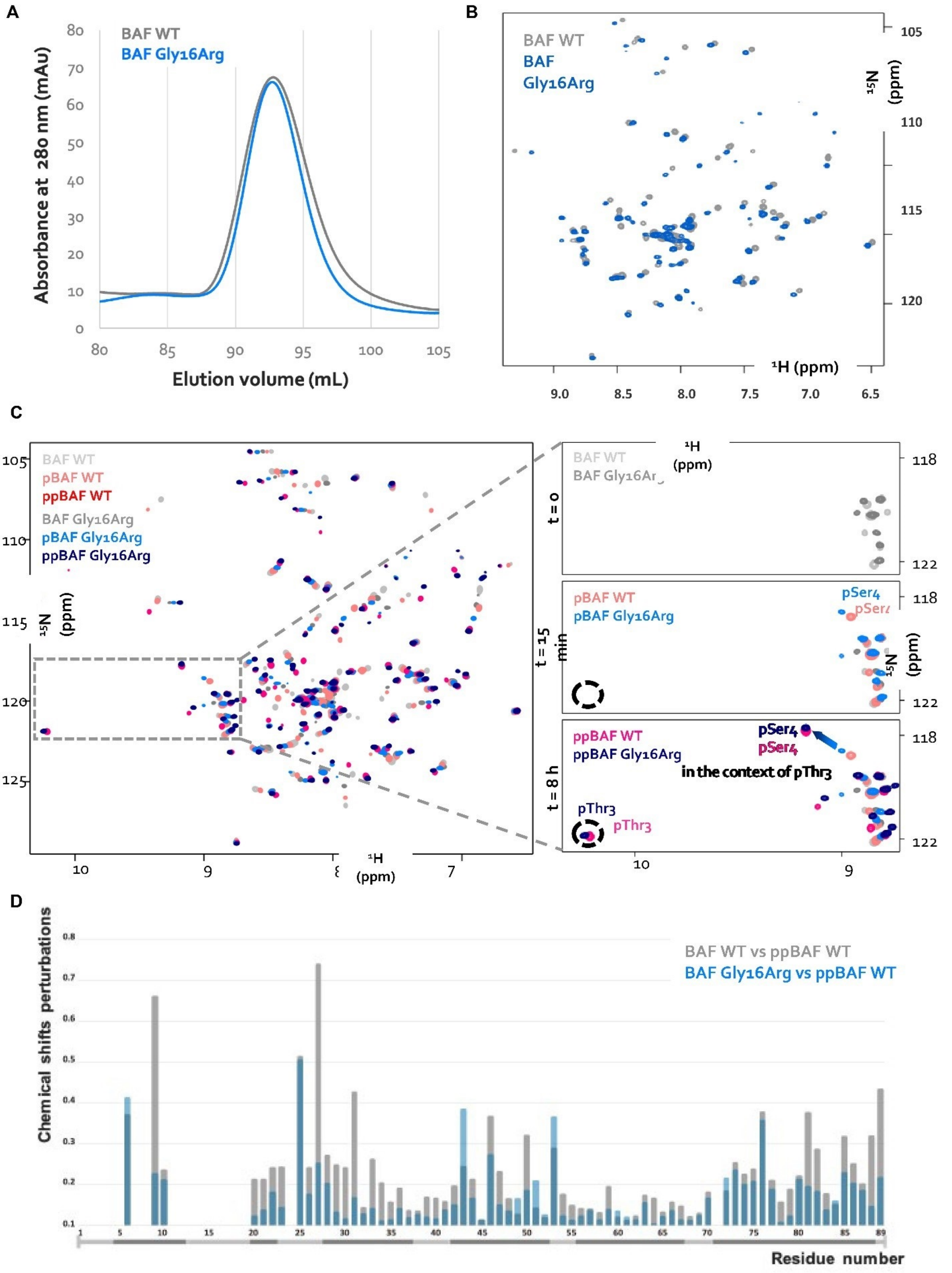

Suppl. Figure S1. Bacterially expressed BAF G16R purifies as a dimer with similar structure and phosphorylation kinetics as compared to BAF WT. A. Size- Exclusion Chromatography profiles of BAF WT (grey) and BAF Gly16Arg (blue), showing that both proteins are eluted as a dimer. B. Superimposition of the 2D NMR 1H-15N HSQC

spectra of BAF WT (grey) and BAF Gly16Arg (blue). In a 1H-15N HSQC NMR spectrum, each peak reports for the chemical environment of a residue. Here the two spectra were recorded in 40 mM sodium phosphate pH 7.2, 150 mM NaCl at 293 K. They are similar, indicating that BAF WT and Gly16Arg share the same fold. C. Superimposition of the 2D NMR 1H-15N HSQC spectra recorded on either BAF WT or BAF Gly16Arg during phosphorylation by VRK1 in the NMR phosphorylation buffer at 303 K. For both proteins, initial spectra are displayed (WT: light grey; Gly16Arg: dark grey), as well spectra obtained after 15 minutes (WT: light pink; Gly16Arg: light blue) and 8 hours (WT: red; Gly16Arg: dark blue) in the presence of VRK1. A change in phosphorylation alters the chemical environment of a residue and induces a shift in the position of the corresponding peak in the spectrum. Peaks corresponding to phosphorylated residues generally have 1H chemical shifts larger than 9 ppm. Zoom views on this spectral region show that after 15 minutes, only Ser4 is phosphorylated in both proteins, whereas after 8 hours, both Thr3 and Ser4 are phosphorylated in these proteins. D. Distance between the 1H-15N NMR peaks of either BAF WT (grey) or Gly16Arg (cyan) and di-phosphorylated BAF WT, plotted as a function of BAF residue number. Spectra were recorded in 40 mM sodium phosphate pH 7.2, 150 mM NaCl, at 293 K. The distance between peaks from two BAF species BAF1 and BAF2, also called chemical shift perturbation, was calculated as the square root of $(\partial \text{Hn BAF1} - \partial \text{Hn BAF2})^2 + \frac{(\partial \text{N BAF1} - \partial \text{N BAF2})^2}{25}$. As shown in this panel, for most residues, the BAF Gly16Arg peak is closer from the di-phosphorylated BAF WT peak, as compared to BAF WT.

A

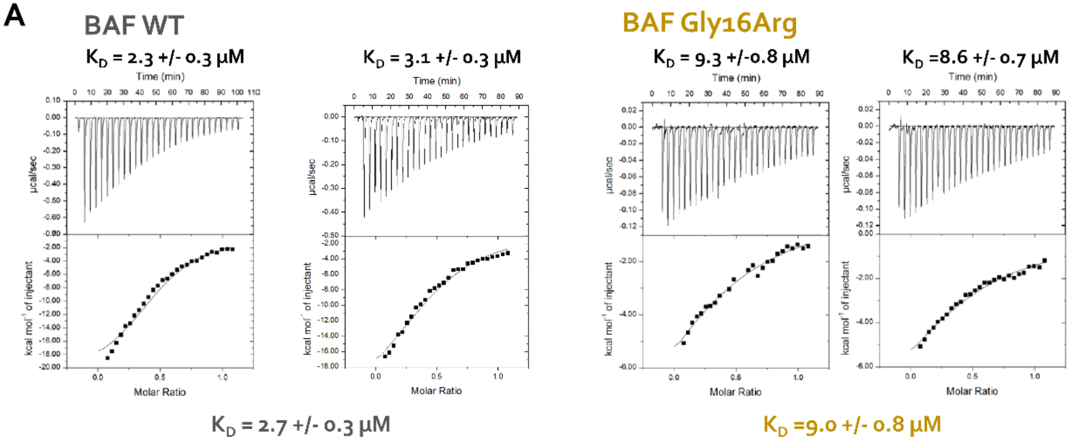

B

Experiment		KD (µM)	KD (µM)	ΔH kcal/mol	ΔH kcal/mol	-TΔS kcal/mol	-TΔS kcal/mol
WT	1	2.3 +/- 0.3	2.7 +/- 0.3	-21.5 +/- 0.7	-23.1 +/- 0.8	13.8	15.4
	2	3.1 +/- 0.3		-24.7 +/- 0.8		17.0	
Gly16Arg	1	9.3 +/- 0.8	9.0 +/- 0.8	-11.6 +/- 0.5	-11.4 +/- 0.5	4.90	4.76
	2	8.6 +/- 0.7		-11.2 +/- 0.4		4.61	

Suppl. Figure S2. BAF Gly16Arg shows reduced binding to the lamin A/C IgFold domain. A. Graphs showing the heat measured during the titrations and the fit performed to obtain the data summarized in B. Binding is measured for BAF WT and Gly16Arg and both experiments are duplicated. Experiment 1 is displayed in Fig. 2B. B. Table summarizing the experimental parameters obtained from the analysis of the ITC curves.

A

5′-CTAGGGTTAGGGTTAGGGTTAG-3′
3′-GATCCCAATCCCAATCCCAATC-5′

B

BAF WT | **BAF Gly16Arg**

K_D = 76 +/- 10 nM | K_D = 158 +/- 27 nM | K_D = 12.3 +/-8.8 nM | K_D =20.3 +/- 14.1 nM

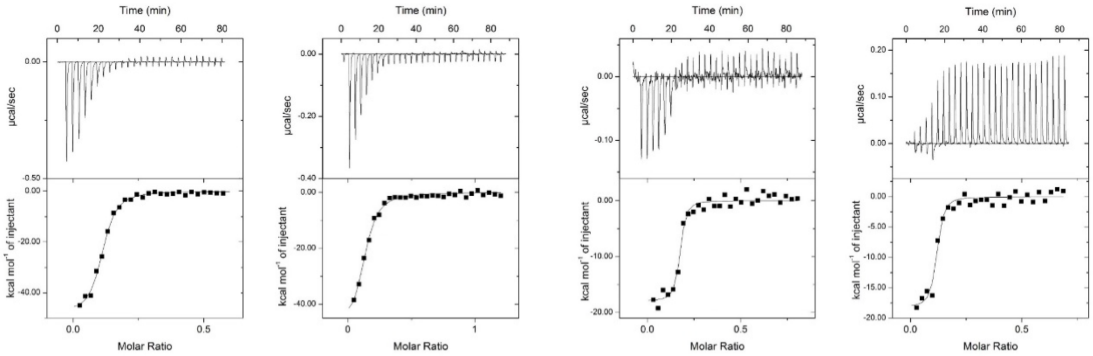

K_D = 117 +/- 58 nM | K_D =16.3 +/- 5.7 nM

C

Experiment		KD (nM)	KD (nM)	ΔH kcal/mol	ΔH kcal/mol	-TΔS kcal/mol	-TΔS kcal/mol
WT	1	76 +/- 10	117 +/- 58	-48,5 +/- 1,0	-49.5 +/- 1,3	39.2	40.4
	2	158 +/- 37		-50,4 +/- 3,5		41.5	
Gly16Arg	1	12.3 +/- 8.8	16.3 +/- 5.7	-18.0 +/- 0.6	-18.1 +/- 0.1	7.52	7.81
	2	20.3 +/- 14.1		-18.2 +/- 0.8		8.09	

Suppl. Figure S3. BAF Gly16Arg shows increased binding to 22 nt-dsDNA relative to BAF WT. A. Sequence of the 22-nt oligonucleotide used in the ITC experiments. B. Graphs showing the heat measured during the titrations and the fit performed to obtain the data summarized in B. Binding is measured for BAF WT and Gly16Arg and both experiments are duplicated. Experiment 1 is displayed in Fig. 5B. C. Table summarizing the experimental parameters obtained from the analysis of the ITC curves.

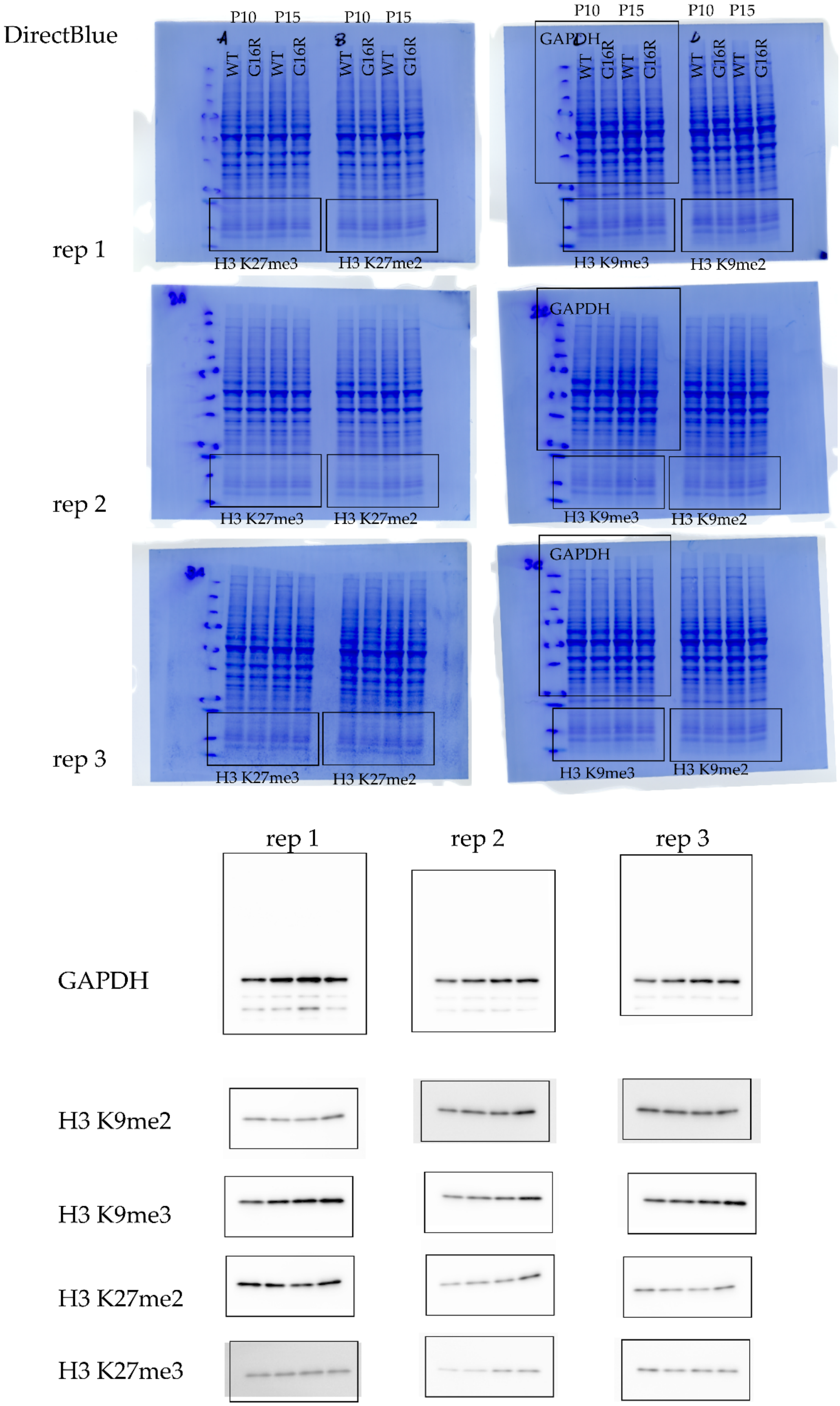

Suppl. Figure S4. Uncropped and unedited images of Western blot membranes used in Figure 5D are shown.
